# Synthesis of Mechanically Robust Very High Molecular Weight Polyisoprene Particle Brushes by Atom Transfer Radical Polymerization

**DOI:** 10.1021/acsmacrolett.4c00089

**Published:** 2024-03-25

**Authors:** Yuqi Zhao, Zongyu Wang, Guanyi Hou, Hanshu Wu, Liye Fu, Michael R. Bockstaller, Xuan Qin, Liqun Zhang, Krzysztof Matyjaszewski

**Affiliations:** † State Key Laboratory of Organic−Inorganic Composites, Beijing University of Chemical Technology, Beijing 100029, China; ‡ Department of Chemistry, Carnegie Mellon University, 4400 Fifth Avenue, Pittsburgh, Pennsylvania 15213, United States; § Department of Materials Science & Engineering, Carnegie Mellon University, 5000 Forbes Avenue, Pittsburgh, Pennsylvania 15213, United States; ∥ College of Chemistry and Materials Engineering, Beijing Technology and Business University, 33th Fucheng Road, Beijing 100048, China

## Abstract

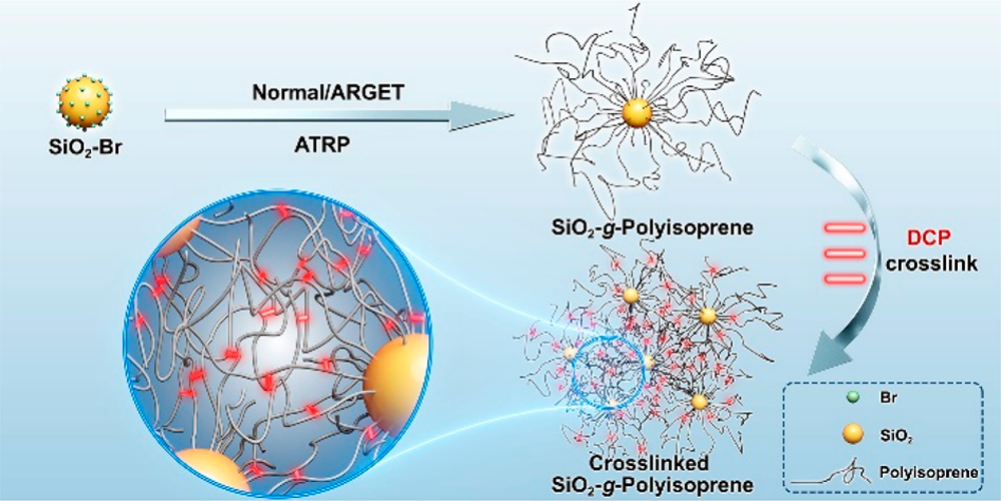

Linear polyisoprene (PI) and SiO_2_-*g*-PI particle brushes were synthesized by both conventional and activators regenerated by electron transfer (ARGET) atom transfer radical polymerization (ATRP). The morphology and solution state study on the particle brushes by transmission electron microscopy (TEM) and dynamic light scattering (DLS) confirmed the successful grafting of PI ligands on the silica surface. The presence of nanoparticle clusters suggests low grafting density (associated with the limited initiation efficiency of ARGET for PI). Nevertheless, particle brushes with very high molecular weights, *M*_n_ > 300,000, were prepared, which significantly improved the dispersion of silica nanoparticles and also contributed to excellent mechanical performance. The reinforcing effects of SiO_2_ nanofillers and very high molecular weight PI ligands were investigated by dynamic mechanical analysis (DMA) as well as computational simulation for the cured linear PI homopolymer/SiO_2_-*g*-PI particle brush bulk films.

Diene monomers (including butadiene, chloroprene, and isoprene) and the resulting polymers and derivative nanomaterials are of particular interest due to their low glass transition temperatures (*T*_g_), excellent degradability, and multifunctionality due to the double-bond-rich composition.^1^ The polymerization of 1,3-diene monomers is a crucial process in both academia and industry fields, with large amounts of commodities being produced.^2,3^ Among the rubber materials, isoprene-based (co)polymers and derivatives are commercially attractive.^4^ Due to their excellent mechanical performance, they form an essential class of elastomers and are widely used in a large range of applications such as adhesives, automotive parts, and medical equipment.^5−7^ Many diene polymers have been prepared through conventional radical polymerization. The use of conventional radical polymerization results in high dispersity, low chain-end functionality, and poor control over the molecular weights, which result in difficulty in preparing block copolymers.^8^ Industrially, polyisoprene has been prepared by alkyllithium-based anionic polymerization,^9^ rare-earth metal-catalyzed polymerization,^10,11^ and Ziegler–Natta coordination polymerization.^12^ Due to the high sensitivity to oxygen, moisture, and impurity, those approaches require extremely harsh reaction conditions such as stringent dry solvents, low reaction temperatures, stringent purification of monomers, etc., which lead to high production costs and limit some polyisoprene applications. Therefore, alternative synthetic methods with low-cost and water-amenable conditions are highly desirable.

In the past few decades, controlled radical polymerization (CRP) has emerged as an advanced technique to tune the molar mass of polymers. Similar to a free radical process, CRP has a high tolerance to a broad range of functionalities, as well as the reaction conditions. Among all CRP techniques, atom transfer radical polymerization (ATRP) has yielded a considerable range of polymeric materials with exceptional control over chain composition, architecture, and functionalities, as well as the morphologies, topologies, and microstructures.^13−15^ Although ATRP has been employed in a vast array of monomers, such as acrylates, methacrylates, acrylamides, styrene, acrylonitrile, etc.,^16−20^ ATRP of isoprene remains challenging. Like all conventional diene-free radical polymerizations, the synthesis of polyisoprene is plagued by drawbacks.^21^ The low monomer boiling point (bp^isoprene^ = 34 °C) demands the use of high-pressure reactor setups.^22^ In addition, due to the resonance structure and equilibrium process of the dominant 1,4-radical, diene allyl radicals proceed with a unique propagation, which results in blends of constitutionally isomeric 1,2-, 3,4-, or 1,4-*cis*/*trans* enchainment with many stereosequences. In contrast to typical monomers (e.g., acrylate, styrene, or acrylonitrile), isoprene has a much lower propagation rate constant (*k*_p_^acrylates^ ≈ 2 × 10^4^ > *k*_p_^styrene^ > 240 *k*_p_^isoprene^ < 100 L M^–1^s ^–1^ at 50 °C),^23,24^ which enables a fairly long reaction time to reach high molecular weight and competition with side reactions, such as Diels–Alder diene dimerization and the allylic poly(diene) hydrogen chain transfer/abstraction, which lead to chain branching or cross-linking at high conversions. There are reports on polymerization of isoprene via nitroxide-mediated polymerization (NMP)^25,26^ and reversible addition–fragmentation chain transfer polymerization (RAFT),^27−29^ while the study on ATRP of isoprene is far from being fully exploited.^30,31^

In this contribution, we explored the preparation of linear PI homopolymer and SiO_2_-*g*-PI particle brushes, applying both conventional and low parts per million levels of Cu catalyst ATRP. Nanosilica is an excellent nanofiller that has been applied in rubber and plastic reinforcement. The bulk aggregation of SiO_2_ nanoparticles in the matrix which is at the lowest energy state is induced by the high surface energy and large surface areas of the nanoparticle, resulting in structural defects in the hybrid nanocomposites and impacting the mechanical properties of the product.^32−34^ The surface properties of the nanosilica are significantly changed by attaching a polymer ligand onto the surfaces of the nanoparticles.^35,36^ The success of grafting the PI polymer ligand onto the silica surfaces was confirmed by transmission electron microscopy (TEM) and dynamic light scattering (DLS). Besides, ultrahigh molecular weight SiO_2_-*g*-PI particle brushes were prepared by ARGET ATRP. The enhancement of tensile strength and toughness in the particle brush system was demonstrated by the computational simulation as well as the dynamic mechanical analysis (DMA) with a comparison of linear PI homopolymer bulk films. Normal ATRP of isoprene was performed using ethyl α-bromoisobutyrate (EBiB) as the initiator and CuBr with an *N*,*N*,*N′*,*N″*,*N*″-pentamethyldiethylenetriamine (PMDETA) ligand as the catalyst system in anisole/DMF under 110 °C (Table 1). Polyisoprene has 4 types of microstructures, namely, *cis*-1,4-polyisoprene, *trans*-1,4-polyisoprene, 1,2-polyisoprene, and 3,4-polyisoprene, due to the different addition modes of isoprene monomers.^37^ Natural rubber is composed of *cis*-1,4-polyisoprene chains with more than 99% *cis*-enchainment, number-average molar masses *M*_n_ of 10^5^–10^6^ g/mol, and molecular weight distribution of 2–10.^38,39^ Polyisoprenes with a rather high *cis*-1,4-content have been synthesized by anionic polymerization. Isoprene can also be polymerized by a radical mechanism in a living fashion but without control of the polymer microstructure: typically, about 80% of 1,4-repeat units (in both *cis*- and *trans*-configurations) and about 20% of 1,2- and 3,4-structures were quantified by NMR spectroscopy.^25,27^ The tacticity of polyisoprene synthesized by ATRP was analyzed by ^1^H NMR and ^13^C NMR (Figure S5), which gives 12.6% 1,2-, 10.4% 3,4-, 29.5% *cis-*1,4-, and 47.5% *trans*-1,4-structures. Tacticity measured by NMR indicated various microstructures along the synthesized polymer chains, which could avoid crystallization of the macromolecules.

**Table 1:** Synthesis of Linear PI Homopolymers and SiO_2_-*g*-PI Particle Brushes

Entry^a^	*M*_n_^b^	*M*_w_/*M*_n_^b^	*f*_ino_ (%)^c^	σ (nm^–2^)^d^
L-1	116,800	2.30	NA	NA
PB-1	97,000	2.56	23	0.12
PB-2	179,000	2.16	32	0.04
PB-3	332,000	1.99	16	0.05

a Reaction conditions. Linear homopolymer: L-1: [isoprene]_0_:[EBiB]_0_:[CuBr]_0_:[PMDETA]_0_ = 1000:1:1:5 in 35 vol % anisole, 15 vol % DMF, 110 °C. Particle brush: PB-1: [isoprene]_0_:[ SiO_2_–Br]_0_:[CuBr]_0_: [PMDETA]_0_ = 20000:1:60:120 in 35 vol % anisole, 15 vol % DMF, 130 °C. PB-2–3: [isoprene]_0_:[SiO_2_–Br]_0_:[CuBr_2_]_0_:[Me6TREN]_0_:[Sn(EH)_2_]_0_ = 20000/30000:1:2:20:20 in 35 vol % anisole, 15 vol % DMF, 110 °C.

b Determined by SEC, Figures S1 and S2.

c The fraction of inorganic content is determined by TGA, Figure S3.

d The grafting density of particle brush is calculated from eq S1 according to TGA data.

Besides the linear PI homopolymer synthesis, 15 nm SiO_2_ nanoparticles were functionalized with an ATRP-initiator-contained anchor, 1-(chlorodimethylsilyl)propyl 2-bromoisobutyrate, following previously reported procedures.^40^ Synthesis of the SiO_2_-*g*-PI particle brush was investigated in the polymerization of isoprene using SiO_2_–Br nanoparticles (Scheme 1). A series of particle brush samples were synthesized according to the formula listed in Table 1. To avoid the gelation, compared to linear homopolymer synthesis, lower initiator concentrations were applied in the particle brush system. However, the reduced initiator concentrations led to a pronounced deceleration of the reaction, yielding a limited conversion rate even when the reaction temperature was elevated to 130 °C. To overcome the limitation, ARGET ATRP of isoprene on the SiO_2_–Br nanoparticle by using tris[2-(dimethylamino)ethyl]amine (Me_6_TREN) instead of PMDETA was further investigated. The Me_6_TREN was selected because compared with PMDETA it is a more active complexing ligand, which is suitable for the low Cu catalyst system. In the ARGET ATRP reaction, by reducing agents, a certain portion of the steady Cu^II^ complex is constantly reduced to highly active Cu^I^ /L species.^41^ One of the benefits of ARGET ATRP is that the amount of Cu catalyst in the reaction can be substantially reduced since Cu^II^/L complexes produced through radical termination (or oxidation reaction) are continuously converted to active Cu^I^/L species by the reducing agent.^24,42,43^ As shown in Table 1, SiO_2_-*g*-PI particle brushes with different chain lengths were prepared with 100 ppm of Cu catalyst. Similar initiation efficiency and molecular weight distribution as the conventional ATRP were observed. An improved dispersion of particle clusters was achieved with longer PI polymer ligands, as shown in the TEM images (Figure 1b and 1c). On the other hand, besides the advantage of introducing a silica core to enhance the mechanical performance of polyisoprene, high molecular weight polymer nanocomposites were synthesized in the particle brushes system. CRP typically yields relatively low molecular weights products (*M*_n_ < 150,000) which result in inferior mechanical performance for the ultimate hybrid materials. Unlike the homogeneous or even distribution of the initiator in the linear homopolymer synthesis system, in the particle brush system, the localization of initiating sites on the surface of nanoparticles considerably changes the reaction dispersion’s homogeneity.^44,45^ In particular, with a larger grafting density, it creates a greater heterogeneity in the system, which leads to less collision and termination between radicals and further results in the synthesis of high molecular weight polymer ligands. Without employing high pressure,^46^ heterogeneous conditions,^47,48^*M*_n_ ∼ 300,000 products were obtained within 8 h of reactions. A better mechanical performance is expected for high molecular weight products. The glass transition temperatures of the SiO_2_-*g*-PI particle brushes were measured by DSC, where the high molecular weight sample gave a slightly higher *T*_g_ (Figure S6).

**Scheme 1:**
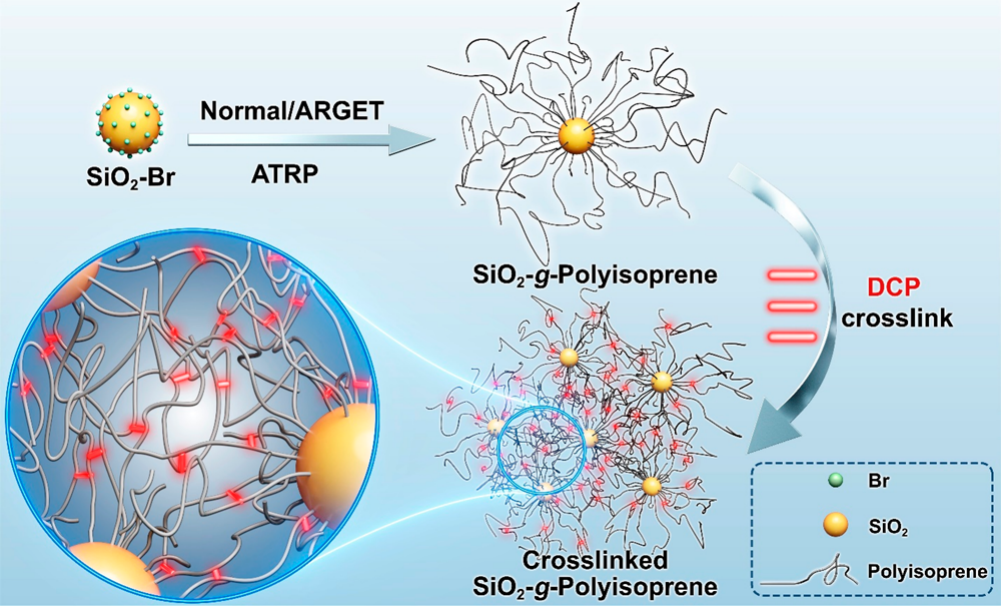
Preparation of SiO_2_-*g*-PI Particle Brushes and Crosslinked PI/SiO_2_ Nanocomposites by Dicumyl Peroxide (DCP)

**Figure 1:**
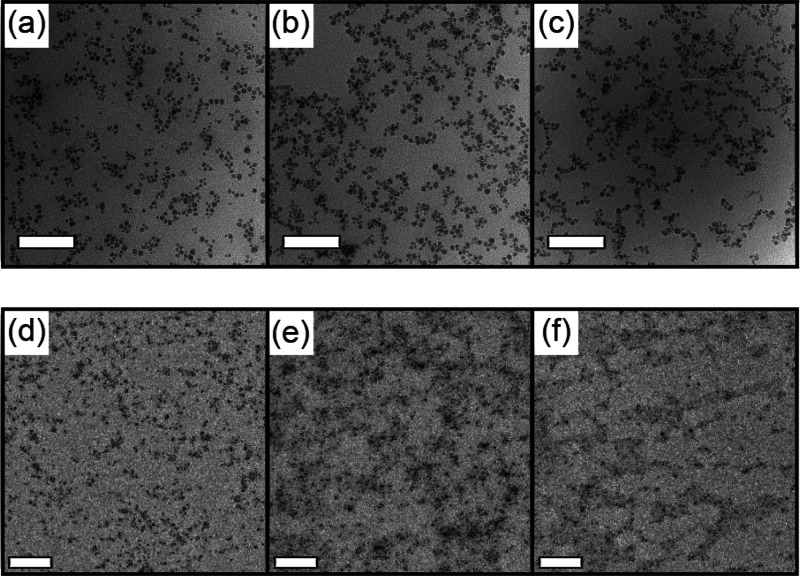
TEM images of SiO_2_-*g*-PI particle brushes: (a) PB-1; (b) PB-2; (c) PB-3. Scale bars: 200 nm. Microsection of SiO_2_-*g*-PI particle brushes: (d) PB-1; (e) PB-2; (f) PB-3. Scale bars: 500 nm.

The microscopic morphology of the monolayer particle brushes prepared via solution casting was characterized by TEM as shown in Figure 1a–c. Obvious small aggregates/clusters shown in Figure 1a suggested a limited density of polymer ligands on the surface and left bare surfaces that induced the grouping of silica nanoparticles as well as the formation of the clusters. This observation aligned with the low apparent grafting density of the particle brush (∼0.1 nm^–2^), and the relatively uniform distribution of the clusters on the Cu grids confirmed the successful grafting of polyisoprene from SiO_2_ nanoparticles.^49^ Interestingly, the morphology of SiO_2_-*g*-PI evolved gradually from small aggregates/clusters structures to string-like structures as the molecular weights increased from 97k (PB-1) to 197k (PB-2) and 332k (PB-3), as depicted in Figure 1b and 1c. This morphology transformation corresponded with the multilayer structures observed in the bulk state, as illustrated by microtome TEM in Figure 1d–f. The presence of fewer nanoparticle aggregates but a more pronounced grayish polymer matrix and the string-like structure in Figure 1f indicated that the long polymer chains in ultrahigh molecular weight SiO_2_-*g*-PI systems formed considerable entanglements, preventing extensive grouping of silica nanoparticles due to the islands of bare surface. The hydrodynamic size of the particle brush dissolved in THF was measured by DLS (Figure S4). The volume-weighted distribution of the particle size (100–400 nm) is consistent with the average value of the clusters determined by TEM.

To elucidate the reinforcing effects of the SiO_2_ nanofillers and molecular weights, linear PI homopolymer and SiO_2_-*g*-PI particle brushes were cured with 5 wt % dicumyl peroxide (DCP) as the cross-linking agent, and uniform bulk films (15 mm × 5 mm, and 0.2 mm in thickness) were fabricated for the mechanical characterizations using uniaxial tensile tests. An observed slight increase in the glass transition temperature (*T*_g_) in both systems indicated the success of the curing process (Figures S6 and S7). Figure 2a presents the stress–strain curves of PI linear homopolymer and particle brushes, with elongation up to 1,000% (due to the limitation of the instrument) at a constant deformation rate of 0.3 s^–1^. The tensile strength and toughness were recorded as the highest point of the curve and calculated by integration of the curve, respectively, as summarized in Figure 2b. As anticipated, the results confirm that introducing stiff SiO_2_ nanoparticles into the nanocomposites increased the tensile strength and toughness. Specifically, compared to cured L-1, cured PB-1 (∼23 wt % silica loading), with a similar molecular weight and dispersity, featured remarkable improvements of 29.3% in tensile strength (from 0.62 to 0.80 MPa) and 79.9% in toughness (from 3.19 MJ/m^3^ to 5.74 MJ/m^3^). We hypothesized that these enhancements are attributed to the strong surface bonding between the SiO_2_ nanoparticles and the polymer ligands, serving as a secondary layer of cross-linking points to reinforce the material.^50,51^ The sheet-like aggregations (PB-2/3) can connect each other via the cross-linked polymer network to form a continuous network, which will promote stress transfer and significantly improve mechanical properties. Furthermore, step-like increases in both tensile strength (1.51 MPA, 88% higher than cured PB-1) and toughness (9.96 MJ/m^3^, 73.5% higher than cured PB-1) were observed in cured PB-3. This significant surge in strength and toughness underlines the substantial contribution of the very high molecular weight of tethered PI polymer chains to the mechanical performance reinforcement. The longer polymer chins formed denser entanglements, as confirmed by microtome TEM in Figure 1f. This not only improved strength but also facilitated the transfer of tension within a polymer chain along its length to numerous other chains, thereby absorbing more energy and resulting in higher toughness.^52,53^

**Figure 2:**
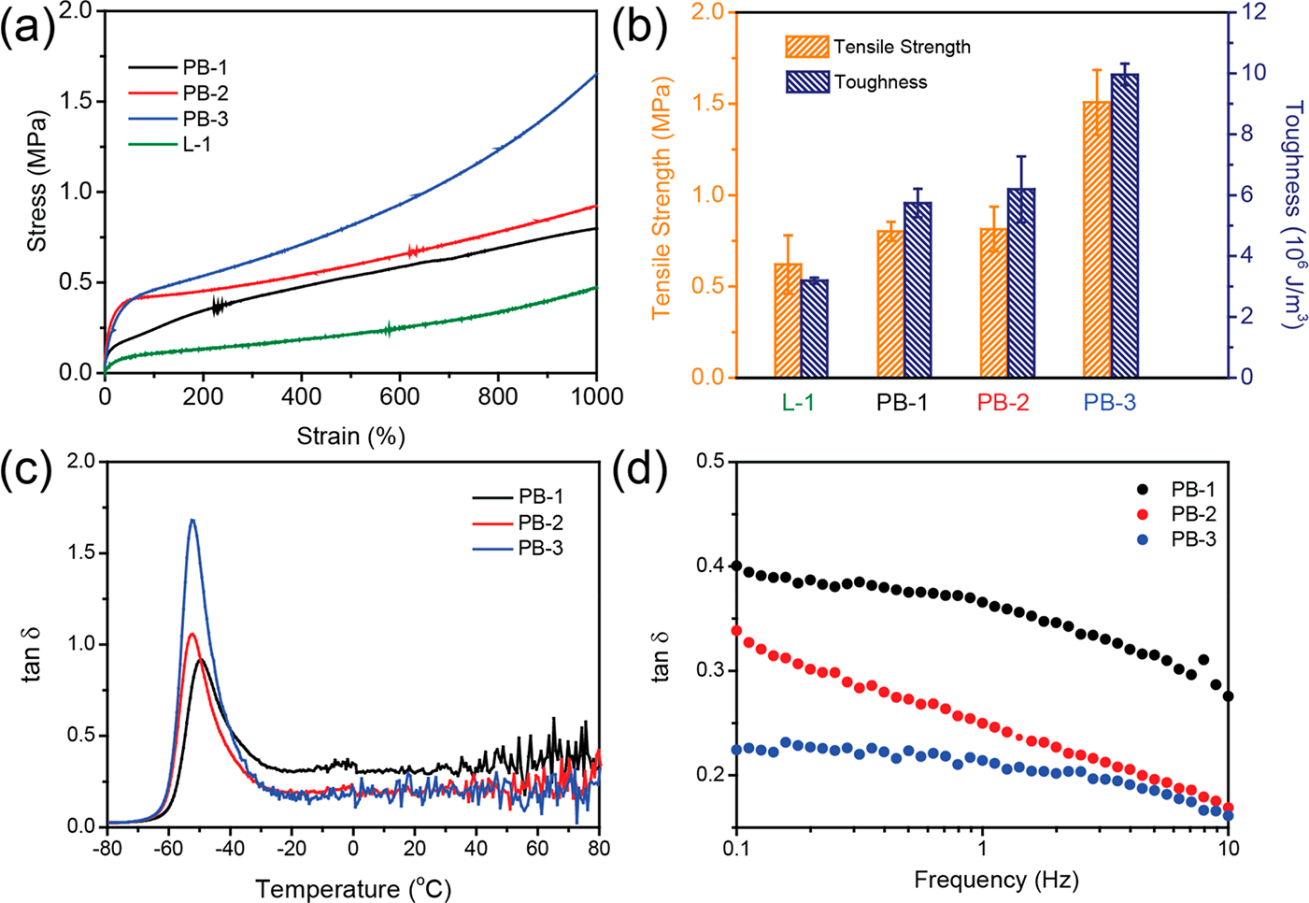
(a) Stress–strain curves of cured PI and SiO_2_-*g*-PI particle brushes. (b) Tensile strength and toughness of cured PI and SiO_2_-*g*-PI particle brushes. (c) Loss factor tan δ for cured SiO_2_-*g*-PI particle brushes. (d) Frequency-dependent tan δ variation for cured SiO_2_-*g*-PI particle brushes. Olive: cured PI. Black: cured PB-1. Red: cured PB-2. Blue: cured PB-3.

Dynamic mechanical analysis (DMA) was applied to further understand the effect of molecular weights on mechanical performances. Figure 2c illustrates the loss tangent (tan δ) for particle brush systems as a function of temperature, providing insights into the viscoelasticity and rolling resistance behavior of the nanocomposites. Similar peak positions indicated comparable glass transition temperatures, consistent with the DSC results. In contrast, higher molecular weights exhibited elevated tan δ peak values (loss modulus/storage modulus), indicating the formation of denser entanglements because of longer tethered chains, resulting in tougher networks for enhanced energy dissipation. For instance, the greater peak value for PB-3 (1.68), compared to those of PB-2 (1.05) and PB-1 (0.92), demonstrated superior energy dissipation ability, aligning with the higher toughness measured during the uniaxial tensile test. Simultaneously, the dense entanglements notably reduced the friction of adjacent nanoparticles, as evidenced by a lower tan δ value at 60 °C, indicating stiffer behavior and reflecting better rolling resistance properties.^54,55^ It is noteworthy that the frequency-dependent tan δ, depicted in Figure 2d, of PB-3 at room temperature was consistently lower than those of PB-1 and PB-2 across the frequency range 0.1–10 Hz. This implies slower chain relaxation and stiffer mechanical behavior.^56^ These improvements suggest that very high molecular weight SiO_2_-*g*-PI might be considered a viable option for potential applications in tire materials or additives.

To further study the dispersion of nanoparticles in the polymer matrix, computational simulations were implemented. The interaction parameter between the matrix and the nanoparticles, ε_np_, was set as 3.5, which was a suitable value to ensure that the spherical nanoparticles would disperse uniformly in the matrix and exhibit relatively better mechanical properties than other values of ε.^57^ The dispersion of nanoparticles on the particle brushes is exhibited in Figure 3(a) to (c), and the radial distribution functions between nanoparticles with different systems were calculated, which are shown in Figure 3d. For PB-1, a pronounced peak appeared at *r* = 3.95σ, which is approximately equal to the diameter of the core nanoparticle that we set, suggesting that some nanoparticles aggregated directly, confirmed by TEM images in Figure 1a and 1d. This is because the number of grafts of PB-1 is small and the core nanoparticle is not completely covered by the graft chain, so the aggregation method is direct contact aggregation.

**Figure 3:**
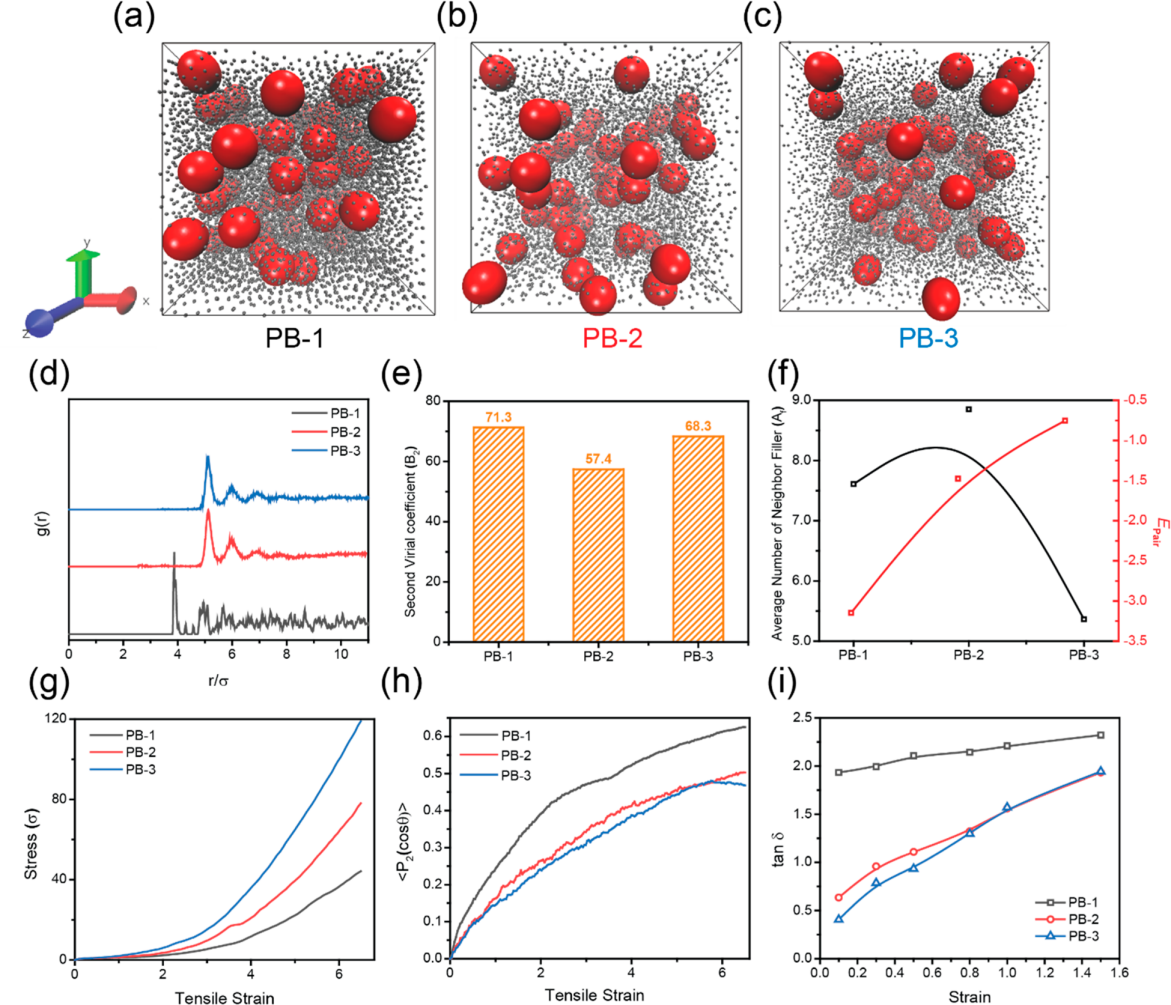
Snapshot of polymer nanocomposites for (a) PB-1, (b) PB-2, and (c) PB-3. The red large spheres denote nanoparticles, and the gray phase represents the grafted polymer chains. (d) The radial distribution functions (RDFs) between nanoparticles with different systems. (e) The second virial coefficient (*B*_2_) and (f) the average number of neighbor filler (*A*_f_) and total interaction energy between filler–filler (*E*_filler–filler_, the right red *Y* axis) with different systems. (g) The orientation of grafted polymer chains, (h) the stress–strain curves with different systems, and (i) the loss factor (tan δ) with different systems.

Based on the results of Figure 3e, the value of B_2_ for PB-1 was positive, meaning that the nanoparticles were dispersed in the polymer chains uniformly. Additionally, the results of the average number of neighbor fillers (*A*_f_, Figure 3f) and total interaction energy between filler–filler (*E*_filler–filler_, Figure 3f) both exhibited that the nanoparticles were well dispersed. These findings suggest that only part of the nanoparticles was aggregated directly, while other nanoparticles were distributed in the simulation box uniformly.^57^

Through the characterization of PB-2 and PB-3, a peak was observed at *r* = 5.12σ for both systems in Figure 3d. These distinct peaks signify that the aggregative modus of nanoparticles was segmental-level tight particle bridging, aligning with the theory of Schweizer.^58^ This result indicated that the nanoparticles were sandwiched between grafted chains. Meanwhile, compared with PB-1, the maximum value of *g*(*r*) was reduced for PB-2 and PB-3, revealing that the dispersion of nanoparticles was highly improved. The same conclusion could also be obtained in Figure 3f, where *E*_filler–filler_ was lower than PB-1.

The entanglement of the grafted polymer chains was characterized using the *Z*1 code to determine the confinement effect.^59−62^ The entanglement network analysis for different polymer nanocomposites is summarized in Table S2. Based on the results, it was observed that the PB-3 obtained the highest value of the average number of entanglements per chain, denoted by ⟨*Z*⟩, which was attributed to the ultrahigh molecular weight ligands.

To investigate the mechanical performance of polymer nanocomposites, uniaxial deformation tests of polymer nanocomposites were carried out. The strain–stress curve for various systems is shown in Figure 3g. The PB-3 has the most remarkable mechanical performance, which corresponds to the dispersion status of the nanoparticles and extensive entanglements. Moreover, the chain orientation behavior during the uniaxial deformation process is also examined by employing the second-order Legendre polynomials (eq S8).^63,64^ The possible values ⟨*P*_2_(cos θ)⟩ range from −0.5 to 1, and the values of −0.5, 1, and 0 indicate an orientation perpendicular to the reference direction, an orientation parallel to the reference direction, or randomly oriented, respectively. Based on the results in Figure 3h, it is observed that for PB-1 the degree of the chain orientation greatly surpassed those of PB-2 and PB-3, indicating that the relatively short chains were easily oriented. In contrast, the very high molecular weight led to denser chain entanglements, elucidating worse chain orientation of PB-3. Because the mechanical performance was attributed to the nanoparticle–polymer chain interaction behavior and the entanglement of grafted polymer chains, the superior mechanical performance of PB-3 suggests that the chain entanglement played a more significant role. This was further confirmed by the alternative variation of the viscoelasticity of the particle brushes shown in Figure 3i. The overall lower tan δ of PB-3 indicates weaker viscosity and stronger elasticity because of denser chain entanglements, exhibiting high fitness with the tendency of experimental DMA results.

In summary, the synthesis of a linear PI homopolymer and SiO_2_-*g*-PI particle brush by conventional ATRP was investigated. The morphology of the particle brush characterized by TEM and DLS confirmed the success of grafting polyisoprene ligands on the surface silica nanoparticles. Small clusters shown on the Cu grids from the aggregation of the particles agreed with the low apparent grafting densities (∼0.1 nm^–2^) of the products. Besides, the low Cu catalyst (100 ppm) ARGET ATRP was applied to synthesize SiO_2_-*g*-PI particle brushes. Compared to linear systems, there is less termination in the particle brush system, which can be applied to synthesize a very high molecular weight polymer nanocomposite. These high molecular weight particle brushes featured extensive entanglements verified by a reduced number of aggregates and the string-like structure observed in microtome TEM images. These nanocomposites hold potential applications in reinforced rubber composites, where the combination of high molecular weight polymer ligands and well-dispersed silica fillers can contribute to excellent mechanical performance. At last, the enhancement of the mechanical performance in particle brush bulk films over the linear PI homopolymers reveals the excellent reinforcing effect of the SiO_2_ nanofillers as well as the strong surface bonding between the nanoparticles and PI polymer ligands, leading to increased particle–polymer interfacial adhesion which was confirmed with both computational and experimental studies.

After this paper was submitted, an interesting report on the synthesis and characterization of silica/PI nanocomposites using RAFT was published.^65^

## Supporting Information Available

Materials and characterization methods; SEC traces for linear PI homopolymers and SiO_2_-*g*-PI particle brushes; TGA, DSC, and DLS curves for SiO_2_-*g*-PI particle brushes; ^1^H NMR and ^13^C NMR spectra for linear polyisoprene; mechanical property measurements; simulation method (PDF)

## References

[ref1] Vasu V.; Kim J.-S.; Yu H.-S.; Bannerman W. I.; Johnson M. E.; Asandei A. D. Normal, ICAR and photomediated butadiene-ATRP with iron complexes. Polym. Chem. 2018, 9 (18), 2389–2406. 10.1039/C8PY00463C.

[ref2] Li W.; Wang H.; Yu L.; Morkved T. L.; Jaeger H. M. Syntheses of Oligophenylenevinylenes–Polyisoprene Diblock Copolymers and Their Microphase Separation. Macromolecules 1999, 32 (9), 3034–3044. 10.1021/ma981679i.

[ref3] Schöps M.; Leist H.; DuChesne A.; Wiesner U. Salt-Induced Switching of Microdomain Morphology of Ionically Functionalized Diblock Copolymers. Macromolecules 1999, 32 (8), 2806–2809. 10.1021/ma9818578.

[ref4] Kongsinlark A.; Rempel G. L.; Prasassarakich P. Hydrogenated polyisoprene-silica nanoparticles and their applications for nanocomposites with enhanced mechanical properties and thermal stability. J. Nanopart. Res. 2013, 15 (5), 1612 10.1007/s11051-013-1612-7.

[ref5] Leber A. P. Overview of isoprene monomer and polyisoprene production processes. Chemico-Biological Interactions 2001, 135–136, 169–173. 10.1016/S0009-2797(01)00189-2. 11397389

[ref6] Nazhat S. N.; Parker S.; Patel M. P.; Braden M. Isoprene–styrene copolymer elastomer and tetrahydrofurfuryl methacrylate mixtures for soft prosthetic applications. Biomaterials 2001, 22 (17), 2411–2416. 10.1016/S0142-9612(00)00428-2. 11511038

[ref7] Bonnevide M.; Jimenez A. M.; Dhara D.; Phan T. N. T.; Malicki N.; Abbas Z. M.; Benicewicz B.; Kumar S. K.; Couty M.; Gigmes D.; Jestin J. Morphologies of Polyisoprene-Grafted Silica Nanoparticles in Model Elastomers. Macromolecules 2019, 52 (20), 7638–7645. 10.1021/acs.macromol.9b01479.

[ref8] Germack D. S.; Wooley K. L. Isoprene polymerization via reversible addition fragmentation chain transfer polymerization. J. Polym. Sci., Part A: Polym. Chem. 2007, 45 (17), 4100–4108. 10.1002/pola.22226.

[ref9] Kamienski C. W. LITHIUM CATALYSIS IN INDUSTRIAL POLYMERIZATION. Industrial & Engineering Chemistry 1965, 57 (1), 38–55. 10.1021/ie50661a007.

[ref10] Gao W.; Cui D. Highly cis-1,4 Selective Polymerization of Dienes with Homogeneous Ziegler–Natta Catalysts Based on NCN-Pincer Rare Earth Metal Dichloride Precursors. J. Am. Chem. Soc. 2008, 130 (14), 4984–4991. 10.1021/ja711146t. 18338895

[ref11] Nishiura M.; Hou Z. Novel polymerization catalysts and hydride clusters from rare-earth metal dialkyls. Nat. Chem. 2010, 2 (4), 257–268. 10.1038/nchem.595. 21124505

[ref12] Tse C. K. W.; Kumar K. R.; Drewitt M. J.; Baird M. C. Isobutene Polymerization and Copolymerization with Isoprene Initiated by [Cp*TiMe2]+ in the Presence of a Novel Type of Weakly Coordinating Counteranion. Macromol. Chem. Phys. 2004, 205 (11), 1439–1444. 10.1002/macp.200400053.

[ref13] Barbey R.; Lavanant L.; Paripovic D.; Schüwer N.; Sugnaux C.; Tugulu S.; Klok H.-A. Polymer Brushes via Surface-Initiated Controlled Radical Polymerization: Synthesis, Characterization, Properties, and Applications. Chem. Rev. 2009, 109 (11), 5437–5527. 10.1021/cr900045a. 19845393

[ref14] Matyjaszewski K. Advanced Materials by Atom Transfer Radical Polymerization. Adv. Mater. 2018, 30 (23), 1706441 10.1002/adma.201706441. 29582478

[ref15] Matyjaszewski K. Architecturally Complex Polymers with Controlled Heterogeneity. Science 2011, 333 (6046), 1104–1105. 10.1126/science.1209660. 21868664

[ref16] Xia J.; Matyjaszewski K. Controlled/“Living” Radical Polymerization. Atom Transfer Radical Polymerization Catalyzed by Copper(I) and Picolylamine Complexes. Macromolecules 1999, 32 (8), 2434–2437. 10.1021/ma981694n.

[ref17] Matyjaszewski K.; Xia J. Atom Transfer Radical Polymerization. Chem. Rev. 2001, 101 (9), 2921–2990. 10.1021/cr940534g. 11749397

[ref18] Matyjaszewski K.; Tsarevsky N. V. Macromolecular Engineering by Atom Transfer Radical Polymerization. J. Am. Chem. Soc. 2014, 136 (18), 6513–6533. 10.1021/ja408069v. 24758377

[ref19] Matyjaszewski K.; Tsarevsky N. V. Nanostructured functional materials prepared by atom transfer radical polymerization. Nat. Chem. 2009, 1 (4), 276–288. 10.1038/nchem.257. 21378870

[ref20] Tsarevsky N. V.; Matyjaszewski K. Green” Atom Transfer Radical Polymerization: From Process Design to Preparation of Well-Defined Environmentally Friendly Polymeric Materials. Chem. Rev. 2007, 107 (6), 2270–2299. 10.1021/cr050947p. 17530906

[ref21] Yu H.-S.; Kim J.-S.; Vasu V.; Simpson C. P.; Asandei A. D. Cu-Mediated Butadiene ATRP. ACS Catal. 2020, 10 (12), 6645–6663. 10.1021/acscatal.0c01207.

[ref22] Weerts P. A.; German A. L.; Gilbert R. G. Kinetic aspects of the emulsion polymerization of butadiene. Macromolecules 1991, 24 (7), 1622–1628. 10.1021/ma00007a027.

[ref23] Domski G. J.; Rose J. M.; Coates G. W.; Bolig A. D.; Brookhart M. Living alkene polymerization: New methods for the precision synthesis of polyolefins. Prog. Polym. Sci. 2007, 32 (1), 30–92. 10.1016/j.progpolymsci.2006.11.001.

[ref24] Ribelli T. G.; Lorandi F.; Fantin M.; Matyjaszewski K. Atom Transfer Radical Polymerization: Billion Times More Active Catalysts and New Initiation Systems. Macromol. Rapid Commun. 2019, 40 (1), 1800616 10.1002/marc.201800616. 30375120

[ref25] Benoit D.; Harth E.; Fox P.; Waymouth R. M.; Hawker C. J. Accurate Structural Control and Block Formation in the Living Polymerization of 1,3-Dienes by Nitroxide-Mediated Procedures. Macromolecules 2000, 33 (2), 363–370. 10.1021/ma991187l.

[ref26] Contreras-López D.; Albores-Velasco M.; Saldívar-Guerra E. Isoprene (co)polymers with glycidyl methacrylate via bimolecular and unimolecular nitroxide mediated radical polymerization. J. Appl. Polym. Sci. 2017, 134 (29), 45108 10.1002/app.45108.

[ref27] Jitchum V.; Perrier S. Living Radical Polymerization of Isoprene via the RAFT Process. Macromolecules 2007, 40 (5), 1408–1412. 10.1021/ma061889s.

[ref28] Bar-Nes G.; Hall R.; Sharma V.; Gaborieau M.; Lucas D.; Castignolles P.; Gilbert R. G. Controlled/living radical polymerization of isoprene and butadiene in emulsion. Eur. Polym. J. 2009, 45 (11), 3149–3163. 10.1016/j.eurpolymj.2009.08.004.

[ref29] Contreras-López D.; Fuentes-Ramírez R.; Albores-Velasco M.; de los Santos-Villarreal G.; Saldívar-Guerra E. Synthesis and characterization of isoprene polymers with polar groups via reversible addition-fragmentation chain-transfer polymerization. J. Polym. Sci., Part A: Polym. Chem. 2018, 56 (21), 2463–2474. 10.1002/pola.29221.

[ref30] Wootthikanokkhan J.; Peesan M.; Phinyocheep P. Atom transfer radical polymerizations of (meth)acrylic monomers and isoprene. Eur. Polym. J. 2001, 37 (10), 2063–2071. 10.1016/S0014-3057(01)00076-3.

[ref31] Wang B.; Wang Z.; Jiang F.; Fang H.; Wang Z. Synthesis and characterization of MWCNT-graft-polyisoprene via ARGET ATRP. RSC Adv. 2014, 4 (50), 26468–26475. 10.1039/c4ra02986k.

[ref32] Gangopadhyay R.; De A. Conducting Polymer Nanocomposites: A Brief Overview. Chem. Mater. 2000, 12 (3), 608–622. 10.1021/cm990537f.

[ref33] Zou H.; Wu S.; Shen J. Polymer/Silica Nanocomposites: Preparation, Characterization, Properties, and Applications. Chem. Rev. 2008, 108 (9), 3893–3957. 10.1021/cr068035q. 18720998

[ref34] Smith W. E.; Zukoski C. F. Aggregation and gelation kinetics of fumed silica–ethanol suspensions. J. Colloid Interface Sci. 2006, 304 (2), 359–369. 10.1016/j.jcis.2006.09.016. 17034807

[ref35] Yu Y.; Rong M. Z.; Zhang M. Q. Grafting of hyperbranched aromatic polyamide onto silica nanoparticles. Polymer 2010, 51 (2), 492–499. 10.1016/j.polymer.2009.12.013.

[ref36] Liu C.-H.; Pan C.-Y. Grafting polystyrene onto silica nanoparticles via RAFT polymerization. Polymer 2007, 48 (13), 3679–3685. 10.1016/j.polymer.2007.04.055.

[ref37] Li J.; Wang J.; Chen H.; Sun B. Influence of Hot Pressing Sintering Temperature on beta-Sialon-15R Ceramics Synthesized from Aluminum Dross. Mater. Trans. 2012, 53 (8), 1495–1501. 10.2320/matertrans.M2012126.

[ref38] Kostjuk S. V.; Ouardad S.; Peruch F.; Deffieux A.; Absalon C.; Puskas J. E.; Ganachaud F. Carbocationic Polymerization of Isoprene Co-initiated by B(C6F5)3: An Alternative Route toward Natural Rubber Polymer Analogues?. Macromolecules 2011, 44 (6), 1372–1384. 10.1021/ma1027966.

[ref39] Puskas J. E.; Gautriaud E.; Deffieux A.; Kennedy J. P. Natural rubber biosynthesis—A living carbocationic polymerization?. Prog. Polym. Sci. 2006, 31 (6), 533–548. 10.1016/j.progpolymsci.2006.05.002.

[ref40] Yan J.; Kristufek T.; Schmitt M.; Wang Z.; Xie G.; Dang A.; Hui C. M.; Pietrasik J.; Bockstaller M. R.; Matyjaszewski K. Matrix-free Particle Brush System with Bimodal Molecular Weight Distribution Prepared by SI-ATRP. Macromolecules 2015, 48 (22), 8208–8218. 10.1021/acs.macromol.5b01905.

[ref41] Ding H.; Park S.; Zhong M.; Pan X.; Pietrasik J.; Bettinger C. J.; Matyjaszewski K. Facile Arm-First Synthesis of Star Block Copolymers via ARGET ATRP with ppm Amounts of Catalyst. Macromolecules 2016, 49 (18), 6752–6760. 10.1021/acs.macromol.6b01597.

[ref42] Kwak Y.; Magenau A. J. D.; Matyjaszewski K. ARGET ATRP of Methyl Acrylate with Inexpensive Ligands and ppm Concentrations of Catalyst. Macromolecules 2011, 44 (4), 811–819. 10.1021/ma102665c.

[ref43] Matyjaszewski K.; Dong H.; Jakubowski W.; Pietrasik J.; Kusumo A. Grafting from Surfaces for “Everyone”: ARGET ATRP in the Presence of Air. Langmuir 2007, 23 (8), 4528–4531. 10.1021/la063402e. 17371060

[ref44] Bevington J. C.; Melville H. W.; Taylor R. P. The termination reaction in radical polymerizations. Polymerizations of methyl methacrylate and styrene at 25°. J. Polym. Sci. 1954, 12 (1), 449–459. 10.1002/pol.1954.120120136.

[ref45] Wang Z.; Liu T.; Lin K. C.; Li S.; Yan J.; Olszewski M.; Sobieski J.; Pietrasik J.; Bockstaller M. R.; Matyjaszewski K. Synthesis of Ultra-high Molecular Weight SiO2-g-PMMA Particle Brushes. Journal of Inorganic and Organometallic Polymers and Materials 2020, 30 (1), 174–181. 10.1007/s10904-019-01289-8.

[ref46] Mueller L.; Jakubowski W.; Matyjaszewski K.; Pietrasik J.; Kwiatkowski P.; Chaladaj W.; Jurczak J. Synthesis of high molecular weight polystyrene using AGET ATRP under high pressure. Eur. Polym. J. 2011, 47 (4), 730–734. 10.1016/j.eurpolymj.2010.10.006.

[ref47] Simms R. W.; Cunningham M. F. High Molecular Weight Poly(butyl methacrylate) by Reverse Atom Transfer Radical Polymerization in Miniemulsion Initiated by a Redox System. Macromolecules 2007, 40 (4), 860–866. 10.1021/ma061899t.

[ref48] Zetterlund P. B.; Kagawa Y.; Okubo M. Controlled/Living Radical Polymerization in Dispersed Systems. Chem. Rev. 2008, 108 (9), 3747–3794. 10.1021/cr800242x. 18729519

[ref49] Lee J.; Wang Z.; Zhang J.; Yan J.; Deng T.; Zhao Y.; Matyjaszewski K.; Bockstaller M. R. Molecular Parameters Governing the Elastic Properties of Brush Particle Films. Macromolecules 2020, 53 (4), 1502–1513. 10.1021/acs.macromol.9b01809.

[ref50] Zhao Y.; Wu H.; Yin R.; Yu C.; Matyjaszewski K.; Bockstaller M. R. Copolymer Brush Particle Hybrid Materials with “Recall-and-Repair” Capability. Chem. Mater. 2023, 35 (17), 6990–6997. 10.1021/acs.chemmater.3c01234. 37719032 PMC10501442

[ref51] Gong J. P.; Katsuyama Y.; Kurokawa T.; Osada Y. Double-Network Hydrogels with Extremely High Mechanical Strength. Adv. Mater. 2003, 15 (14), 1155–1158. 10.1002/adma.200304907.

[ref52] Kim J.; Zhang G.; Shi M.; Suo Z. Fracture, fatigue, and friction of polymers in which entanglements greatly outnumber cross-links. Science 2021, 374 (6564), 212–216. 10.1126/science.abg6320. 34618571

[ref53] Steck J.; Kim J.; Kutsovsky Y.; Suo Z. Multiscale stress deconcentration amplifies fatigue resistance of rubber. Nature 2023, 624 (7991), 303–308. 10.1038/s41586-023-06782-2. 38092910

[ref54] Liu J.; Zheng Z.; Li F.; Lei W.; Gao Y.; Wu Y.; Zhang L.; Wang Z. L. Nanoparticle chemically end-linking elastomer network with super-low hysteresis loss for fuel-saving automobile. Nano Energy 2016, 28, 87–96. 10.1016/j.nanoen.2016.08.002.

[ref55] Qin X.; Wang J.; Zhang Y.; Wang Z.; Li S.; Zhao S.; Tan T.; Liu J.; Zhang L.; Matyjaszewski K. Self-Assembly Strategy for Double Network Elastomer Nanocomposites with Ultralow Energy Consumption and Ultrahigh Wear Resistance. Adv. Funct. Mater. 2020, 30 (34), 2003429 10.1002/adfm.202003429.

[ref56] Zhao Y.; Wang Z.; Yu C.; Wu H.; Olszewski M.; Yin R.; Zhai Y.; Liu T.; Coronado A.; Matyjaszewski K.; Bockstaller M. R. Topologically Induced Heterogeneity in Gradient Copolymer Brush Particle Materials. Macromolecules 2022, 55 (19), 8846–8856. 10.1021/acs.macromol.2c01131.

[ref57] Hou G.; Tao W.; Liu J.; Gao Y.; Zhang L.; Li Y. Tailoring the dispersion of nanoparticles and the mechanical behavior of polymer nanocomposites by designing the chain architecture. Phys. Chem. Chem. Phys. 2017, 19 (47), 32024–32037. 10.1039/C7CP06199D. 29181472

[ref58] Hooper J. B.; Schweizer K. S. Theory of Phase Separation in Polymer Nanocomposites. Macromolecules 2006, 39 (15), 5133–5142. 10.1021/ma060577m.

[ref59] Kröger M. Shortest multiple disconnected path for the analysis of entanglements in two- and three-dimensional polymeric systems. Comput. Phys. Commun. 2005, 168 (3), 209–232. 10.1016/j.cpc.2005.01.020.

[ref60] Shanbhag S.; Kröger M. Primitive Path Networks Generated by Annealing and Geometrical Methods: Insights into Differences. Macromolecules 2007, 40 (8), 2897–2903. 10.1021/ma062457k.

[ref61] Hoy R. S.; Foteinopoulou K.; Kröger M. Topological analysis of polymeric melts: Chain-length effects and fast-converging estimators for entanglement length. Phys. Rev. E 2009, 80 (3), 031803 10.1103/PhysRevE.80.031803. 19905139

[ref62] Karayiannis N. C.; Kröger M. Combined Molecular Algorithms for the Generation, Equilibration and Topological Analysis of Entangled Polymers: Methodology and Performance. International Journal of Molecular Sciences 2009, 10 (11), 5054 10.3390/ijms10115054. 20087477 PMC2808023

[ref63] Hou G.; Li S.; Liu J.; Weng Y.; Zhang L. Designing high performance polymer nanocomposites by incorporating robustness-controlled polymeric nanoparticles: insights from molecular dynamics. Phys. Chem. Chem. Phys. 2022, 24 (5), 2813–2825. 10.1039/D1CP04254H. 35043809

[ref64] Moyassari A.; Gkourmpis T.; Hedenqvist M. S.; Gedde U. W. Molecular dynamics simulation of linear polyethylene blends: Effect of molar mass bimodality on topological characteristics and mechanical behavior. Polymer 2019, 161, 139–150. 10.1016/j.polymer.2018.12.012.

[ref65] Dhara D.; Dhara D.; Rahman M. A.; Ruzicka E.; Karekar A.; Vlassopoulos D.; Saalwächter K.; Benicewicz B.; Kumar S. K. Mechanical properties of polyisoprene-based elastomer composites. Macromolecules 2024, 57 (4), 1448–1460. 10.1021/acs.macromol.3c01084.

