# Supplementary material for: Synthesis of Mechanically Robust Very High Molecular Weight Polyisoprene Particle Brushes by Atom Transfer Radical Polymerization
Source: ACS Macro Lett. 2024 Mar 25;13(4):415–22. doi: 10.1021/acsmacrolett.4c00089 (PMC11025114; doi:10.1021/acsmacrolett.4c00089)
Supplement: Supplementary file 1 — mz4c00089_si_001.pdf [file mz4c00089_si_001.pdf]

Supporting Information

Synthesis of Mechanically Robust Very High Molecular Weight Polyisoprene Particle Brushes by ATRP

Yuqi Zhao[⊥,§], *Zongyu Wang*[‡,§], *Guanyi Hou*[#,*], *Hanshu Wu*[⊥], *Liye Fu*[‡], *Michael R. Bockstaller*[⊥], *Xuan Qin*[†,‡,*], *Liqun Zhang*[†], *Krzysztof Matyjaszewski*[‡,*]

[†] State Key Laboratory of Organic-Inorganic Composites, Beijing University of Chemical Technology, Beijing 100029, China

[‡] Department of Chemistry, Carnegie Mellon University, 4400 Fifth Avenue, Pittsburgh, Pennsylvania 15213, United States

[⊥] Department of Materials Science & Engineering, Carnegie Mellon University, 5000 Forbes Avenue, Pittsburgh, Pennsylvania 15213, United States

[#] College of Chemistry and Materials Engineering, Beijing Technology and Business University, 33th Fucheng Road, Beijing 100048, China

[§] Y.Z. and Z.W. contributed equally.

Corresponding Author

* E-mail: hougy@btbu.edu.cn
* E-mail: qinxuan@mail.buct.edu.cn
* E-mail: matyjaszewski@cmu.edu

Experimental Section

Materials.

Monomer: isoprene (I, 99%, Aldrich), and methyl methacrylate (MMA, 99%, Aldrich) were purified by passing through a column filled with alumina to remove the inhibitor. Copper (I) bromide (CuBr, 98%, Acros), was washed with glacial acetic acid to remove any soluble oxidized species, filtered, washed twice with anhydrous ethyl ether, dried, and kept under vacuum. Tris(2-dimethylaminoethyl)amine (Me_6TREN, 99%, Alfa), tetrahydrofuran (THF, 99%, VWR), anisole (99%, Aldrich), *N,N,N',N'',N''*-pentamethyldiethylenetriamine (PMDETA, 99%, Aldrich), ethyl 2-bromoisobutyrate (EBiB, 98%, Aldrich), methanol (99%, VWR), hexane (99%, VWR), acetone (99%, VWR), *N*,*N*-dimethylformamide (DMF, 99%, VWR), copper(II) bromide ($CuBr_2$, 99%, Aldrich), tin(II) 2-ethylhexanoate ($Sn(EH)_2$, 95%, Aldrich), 48% hydrofluoric acid aqueous solution (HF, >99.99%, Aldrich), ammonium hydroxide aqueous solution (NH_4OH, 28.0-30.0%, Fisher), anhydrous magnesium sulfate ($MgSO_4$, Fisher), allyl alcohol (99%, Aldrich, 99%), triethylamine (TEA, 99.5%, Aldrich), 2-bromoisobutyryl bromide (2BiB, 98%, Aldrich), platinum(0)-1,3-divinyl-1,1,3,3- tetramethylsiloxane complex in xylene (Karstedt's catalyst, 2% Pt, Aldrich), chlorodimethylsilane (98%, Aldrich), triethoxysilane (95%, Aldrich), hexamethyldisilazane (HMDZ, 99%, Aldrich), dicumyl peroxide (DCP, 98%, Sigma Aldrich) were used as received without further purification. Silica nanoparticles, 30 wt % solution in methyl isobutyl ketone (MIBK-ST), effective diameter $d \approx 15.8$ nm, were kindly donated by Nissan Chemical Corp. and used as received. The tetherable ATRP initiator 1-(chlorodimethylsilyl)propyl-2-bromoisobutyrate and surface-modified silica (SiO_2-Br) were prepared using previously reported procedures. [1-3]

Procedures.

Procedures for the synthesis of linear polyisoprene by normal ATRP.

Initiator (EBiB), solvents (anisole, DMF), and PMDETA were mixed thoroughly in a sealed Ace Glass pressure tube. Purified isoprene was prepared in a separate round flask. Both mixtures were degassed by nitrogen purging, then the isoprene monomer and CuBr powder were added into the Ace Glass pressure tube to activate the catalyst complex, and the tube was immediately sealed and put into an oil bath set at the desired temperature. The reaction mixture was cooled to room

temperature and added to cold methanol to precipitate the product. The molecular weight of the polymer was measured by SEC.

Procedures for the synthesis of SiO_2-g-PI particle brushes by ARGET ATRP.

Macroinitiator (SiO_2-Br nanoparticles), solvents (anisole, DMF), $CuBr_2$, and Me_6TREN were mixed thoroughly in a sealed Ace Glass pressure tube. A stock solution of $Sn(EH)_2$ in anisole and purified isoprene in a round flask were prepared, separately. Both mixtures and the isoprene monomers were degassed by nitrogen purging, then the isoprene monomer and the $Sn(EH)_2$ solution were added into the Ace Glass pressure tube to activate the catalyst complex, and the tube was immediately sealed and put into an oil bath set at the desired temperature. The reaction mixture was cooled to room temperature and added to cold methanol to precipitate the product. The molecular weight of the polymer was measured by SEC.

Procedures for fabrication of linear PI homopolymer and SiO_2-g-PI particle brush bulk films.

Linear PI homopolymers (L-1) and SiO_2-*g*-PI particle brushes (PB-1, PB-2, PB-3) were dispersed in THF *via* sonication. 5 wt% of dicumyl peroxide (DCP) as a crosslinking agent was added to the solution. After the solution was stirred for 0.5 h, the bulk dispersions were transferred into 15 mm × 5 mm rectangular Teflon molds. The solvent was slowly evaporated over 48 h at room temperature generating transparent nanocomposite films with a thickness of 0.15-0.2 mm. The bulk films were transferred to the oven and cured under 160 °C in the N_2 atmosphere for 20 minutes. The residual solvent was removed from the bulk films by transferring them to a vacuum oven and slowly increasing the temperature at the rate of 10 °C per 24 h to 120 °C. At least three specific bulk films of the same composition were investigated to systematically study the thermo-mechanical properties of the nanocomposite films.

Characterization.

Size Exclusion Chromatography (SEC). Number-average molecular weights (M_n) and molecular weight distributions (M_w/M_n) were determined by SEC. The SEC was conducted with a Waters 515 pump and a Waters 410 differential refractometer with PSS columns (Styrogel 105, 103, and 102 Å) in THF as an eluent at 35 °C and a flow rate of 1 mL min^{-1}. Linear PI and PS standards were used for calibration.

Nuclear Magnetic Resonance (NMR). Polymerization was monitored by ^{1}H NMR with a Bruker Advance 500 MHz NMR spectroscopy in $CDCl_3$ solvent. The tacticity of polyisoprene was studied by ^{13}C NMR with a Bruker Advance 500 MHz NMR spectroscopy in $CDCl_3$ solvent.

Transmission Electron Microscopy (TEM). TEM was carried out using a JEOL 2000 EX electron microscope operated at 200 kV to characterize the morphology and structure of the SiO_2-*g*-PI particle brushes, samples were drop cast onto a carbon-coated copper grid. The spatial distribution, radius, and inter-particle distances of the SiO_2 nanoparticles were determined from statistical analysis of the TEM micrographs using ImageJ software. TEM was carried out using a Tecnai G2 F30 electron microscope operated at an acceleration voltage of 300 kV to characterize the morphology and distribution of silica of the cured composite samples. Before characterization, an ultramicrotome (Leica EMUC7) was used to make a nanosheet and a freshly cut surface at -65 °C.

Dynamic Light Scattering (DLS). DLS using a Malvern Zetasizer Ultra was employed to determine the number-weighted average hydrodynamic radius and distribution. The SiO_2-*g*-PI particle brushes were suspended in filtered THF (450 nm PTFE filter) at low concentrations (1 mg mL^{-1}).

Thermogravimetric Analysis (TGA). TGA with TA Instruments 2950 was used to measure the fraction of SiO_2 in the SiO_2-*g*-PI particle brushes. The data were analyzed with TA Universal Analysis. The heating procedure involved four steps: (1) jump to 120 ºC; (2) hold at 120 ºC for 10 min; (3) ramp up at a rate of 20 ºC/min to 800 ºC; (4) hold for 2 min. The TGA plots were normalized to the total weight after holding at 120 ºC.
The grafting density was calculated using the formula (S1).

$$\sigma_{\mathrm{TGA}} = \frac{(1-f_{\mathrm{SiO2}})N_{\mathrm{Av}}\,\rho_{\mathrm{SiO2}}\,d}{6\,f_{\mathrm{SiO2}}\,M_n} \qquad \text{(eq. S1)}$$

where f_{SiO2} is the SiO_2 fraction measured by TGA, N_{Av} is the Avogadro number, ρ_{SiO2} is the density of SiO_2 nanoparticles (2.2 g/cm^3), d is the average diameter of SiO_2 nanoparticles (15.8 nm), M_n is the overall number-average MW of the cleaved polymer brushes.

Differential scanning calorimetry (DSC). The glass transition temperature (T_g) of linear PI homopolymers and SiO_2-*g*-PI particle brushes were measured by differential scanning calorimetry (DSC) with TA Instrument QA-2000. The same procedure was run three times, each involving the

following steps: (1) Equilibrate at 25.00 °C, (2) Isothermal for 1.00 min, (3) Ramp 20.00 °C/min to -90.00 °C, (4) Isothermal for 1.00 min, (5) Ramp 20.00 °C/min to 80.00 °C, (6) Isothermal for 1.00 min, (7) Ramp 20.00 °C/min to -90.00 °C, (8) Isothermal for 1.00 min, (9) Ramp 20.00 °C/min to 80.00 °C, (10) Isothermal for 1.00 min, (11) Ramp 20.00 °C/min to -90.00 °C, (12) Isothermal for 1.00 min, (13) Ramp 20.00 °C/min to 80.00 °C, (14) Isothermal for 1.00 min, (15) Jump to 25.00 °C. The DSC data were analyzed with a TA Universal Analysis instrument, and T_g was directly acquired.

Dynamic Mechanical Analysis (DMA).

Tensile test: the linear PI homopolymers (L-1) and SiO_2-*g*-PI (PB-1, PB-2, PB-3) particle brush bulk films are tested in the tensile mode by using DMA (TA RSA-G2). The film thickness was between 150-200 μm. The samples were stretched at a constant tensile rate of 0.3 s^{-1} at room temperature.

Glass transition temperature measurement: The glass transition temperature was also measured through dynamic mechanical analysis (DMA, TA RSA-G2) at a constant frequency (1 Hz) in a temperature range of -80 ℃ to 80 ℃, with a heating rate of 3 ℃/min, and application of 0.1% strain.

Damping property measurement: The damping property was measured through dynamic mechanical analysis (DMA, TA RSA-G2) in a frequency range of 0.1-10 Hz at room temperature, with the application of 0.1% strain. All the samples were tested at least three times for consistency.

Determining the initiating site density for SiO_2-Br nanoparticles. The concentration of initiating sites on the surface of silica nanoparticles was determined by model reactions (i.e. polymerization of SiO_2-*g*-PMMA, $[MMA]_0/[SiO_2\text{-}Br]_0/[CuBr_2]_0/[Me_6TREN]_0/[Sn(EH)_2]_0$ = 2000:1:1:10:8 with 45 vol% anisoles, 5 vol% DMF at 60 ºC) with certain amount of SiO_2-Br nanoparticles (e.g. 100 mg). After purification, SEC and TGA were conducted to characterize the grafting density of the particle brushes. The particles used in the current study had grafting density (σ_0) ~ 0.15 nm^{-2}, the -Br (initiating site) concentration on the surface was assumed the same. Based on the average radius of nanoparticles, 7.9 nm, and density of silica, 2.2 g/cm^3, the average molar mass of SiO_2-Br, is 23,270 g/mol.

Simulation Method.

In this study, the coarse-grain MD simulation was used to study the effect of the structure of the nanoparticles. In this simulation model, different numbers of grafted chains with various chain lengths (D = 1σ) are grafted on the core nanoparticle (D = 4σ). (Scheme S1). To correspond to the experimental section. The grafted amount (N_g) and length (L) of grafted chains are specific, which is shown in Table S1. To allow the modified nanoparticle to reach maximum grafted density, based on Thomson's theory,[4] a numerical method was adopted: a set of points with random velocity and coordinates are constrained on a spherical surface, and then the average distance of the adjacent particles are calculated until the system becomes stable. Iterating the process, the maximum number of points is achieved when the average distance is equal to the diameter of the surficial particles. The maximum number of grafted points is calculated by the following equation:

$$d = < H(r_i, r_j) > \qquad \text{(eq. S2)}$$

where d is the average distance of the adjacent particles and r_i/r_j represents particle i and its neighbor j. H is the nearest neighbor function measured by Euclidean distance.

Scheme S1. The schematic diagram of PB-1, PB-2, and PB-3, where red spheres represented the nanoparticle, and gray spheres represented the grafted polymer chains with different lengths.

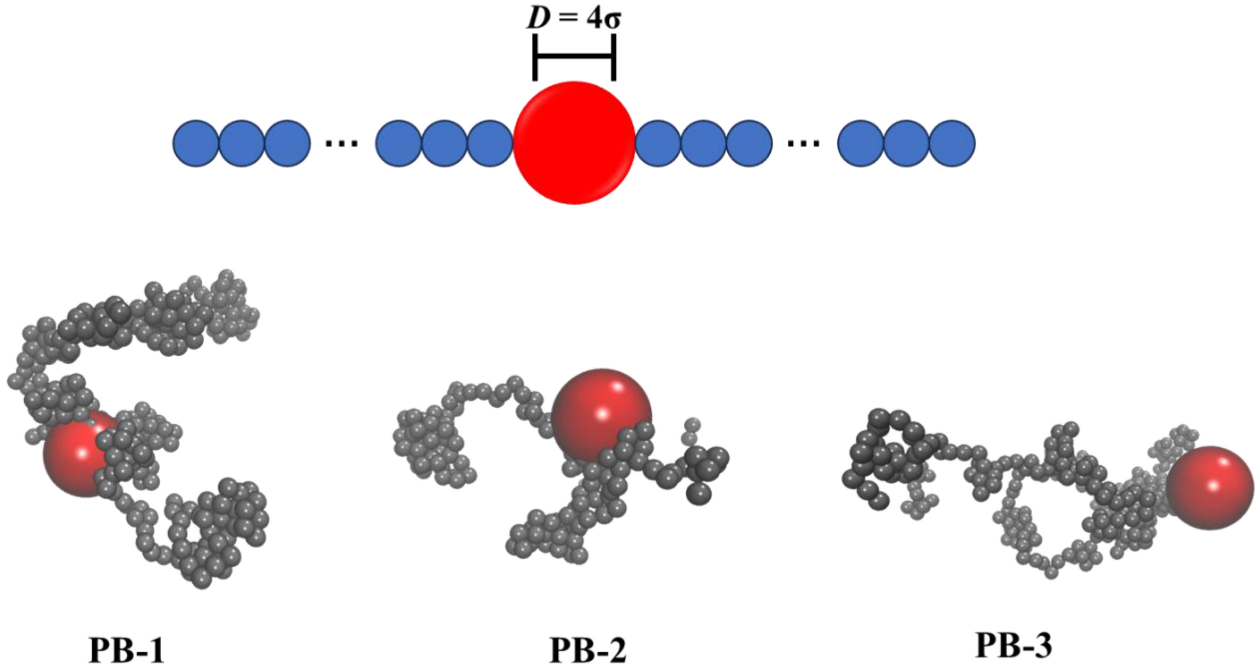

Table S1. The modeling details of grafted nanoparticles.

system	grafted amount (N_g)	length (L)
PB-1	6	41σ
PB-2	2	76σ
PB-3	2	144σ

The typical Lennard-Jones (LJ) potential was employed to model the interaction between different types of beads. The formula of the potential is:

$$U_{ij}(r)=\begin{cases}4\varepsilon_{ij}\left[\left(\dfrac{\sigma}{r-r_{EV}}\right)^{12}-\left(\dfrac{\sigma}{r-r_{EV}}\right)^{6}\right]\text{-}U_{cutoff} & 0<r-r_{EV}<r_{cutoff}\\ 0 & r-r_{EV}>r_{cutoff}\end{cases} \quad \text{(eq. S3)}$$

where r_{cutoff} denotes the distance at which the interaction was truncated and shifted so that the energy and force were zero. Here the interaction range was offset by r_{EV} to eliminate the excluded volume effect of different interaction sites. The interaction parameter (ε_{np}) between nanoparticle-matrix is set as 3.5 to endow a measure of the interaction between the nanoparticle and matrix to ensure the dispersion of nanoparticle is uniform,[5] while other ε is set as 1.0.

The bond energy between the connected beads in a polymer chain is represented by a stiff harmonic potential:

$$U_{bond} = k(r - r_0)^2 \qquad \text{(eq. S4)}$$

the bond is set as $k = 500(\varepsilon / \sigma^2)$, r_0 = 2.5, 1.12, and 1.0, corresponding to the 3 types of bonds: core nanoparticle—grafted chain bead, crosslinked bonds, and other bonds. This setting ensured a certain stiffness of the bonds and avoided high-frequency modes and chain crossing. The chemical cross-linking bonds are generated by a random bonding algorithm, ensuring that all cross-linking bonds will not be generated on the same molecular chain. The number of chemical crosslinking bonds is set as 400.[6]

As the grafted polymer is not specified, the reduced LJ units ε and σ are used and set to unity, which means that all calculated quantities are dimensionless. In this study, the reduced units of the temperature and the pressure are adopted and defined as follows:

$$T^* = (k_B T / \varepsilon) \qquad \text{(eq. S5)}$$

$$P^* = P \cdot \sigma^3 / \varepsilon \qquad \text{(eq. S6)}$$

where k_B is the Boltzmann constant which is equal to 1 for LJ potential. The simulations have been performed under the NPT ensemble where the temperature is fixed at T^* = 1.0 and P^* = 1.0 by using the Nose−Hoover temperature thermostat and barostat, respectively. Periodic boundary conditions are employed in all three directions. The velocity-Verlet algorithm is used to integrate the equations of motion, with a time step δ_t = 0.001, where the simulation time is represented by the reduced LJ time τ. All structures are equilibrated over a long time so that each chain has moved at least $2R_g$, where R_g is the root mean square radius of gyration of polymer matrix chains. These fully equilibrated configurations are further used as starting structures for production runs during the structural and dynamic analysis. The Second Virial coefficient (B_2) of the nanoparticles is also calculated, which is a good indicator of the tendency for NPs to aggregate or disperse, given by:

$$B_2 = -\frac{1}{2}\int_0^{\infty} [g(r) - 1] 4\pi r^2 dr \qquad \text{(eq. S7)}$$

$$< P_2(\cos\theta) >= \frac{1}{2}[3(\cos^2\theta) - 1] \qquad \text{(eq. S8)}$$

where θ denotes the angle between a given element (two adjoining monomers in the chains) and the reference stretching direction.

The uniaxial tensile deformation has been performed using the protocol implemented in our previous work.[7] The box length along the z direction is increased at a constant engineering strain rate, while the box lengths along the x and y directions are reduced simultaneously, to maintain the constant volume of the simulation box. The engineering strain rate is specified as $\dot{\varepsilon}_1 = (L(t)_z - L_z)/L_z = 0.0327\tau^{-1}$. The average stress s in the z direction was obtained from the deviated part of the stress tensor: $\sigma_1 = (1+\mu)(-P_{zz}+P) \approx 3(-P_{zz}+P)/2$, whereas $P = \sum_i P_{ii}/3$ was the hydrostatic pressure. The parameter m is Poisson's ratio, which is equal to 0.5 in the present simulations.

Table S2. The entanglement network analysis of polymer nanocomposites with various systems

Entry	$\sqrt{R^2}$	L_p	**< Z >**
PB-1	8.927	13.405	1.460
PB-2	10.194	12.040	2.420
PB-3	13.748	18.156	3.053

For the oscillatory shear deformation, the SLLOD equation of motion and the Lees–Edwards "sliding brick" boundary condition, were adopted. The upper XY plane of the simulation box was shifted along the x direction so that each point in the simulation box could be considered as having a ''streaming'' velocity. This position-dependent steaming velocity was subtracted from the actual velocity of each atom to yield a thermal velocity, which could be used for the temperature computation thermosetting. The shear strain was defined as $\gamma = \delta_x / L_z(0)$, where the offset dX was the transverse displacement distance in the shear direction from the unstrained orientation, and $L_Z(0)$ was the box length perpendicular to the shear direction. The shear rate, n, was set as 0.01/τ, showing one cycle of constant-amplitude oscillation every 100t. The average shear stress was obtained from the deviatoric part of the stress tensor: $\delta_s = P_{XY} = P_{YX}$.

All the MD simulations were carried out using the Largescale Atomic/Molecular Massively Parallel Simulator (distributed by Sandia National Laboratories).[8]

Supporting data:

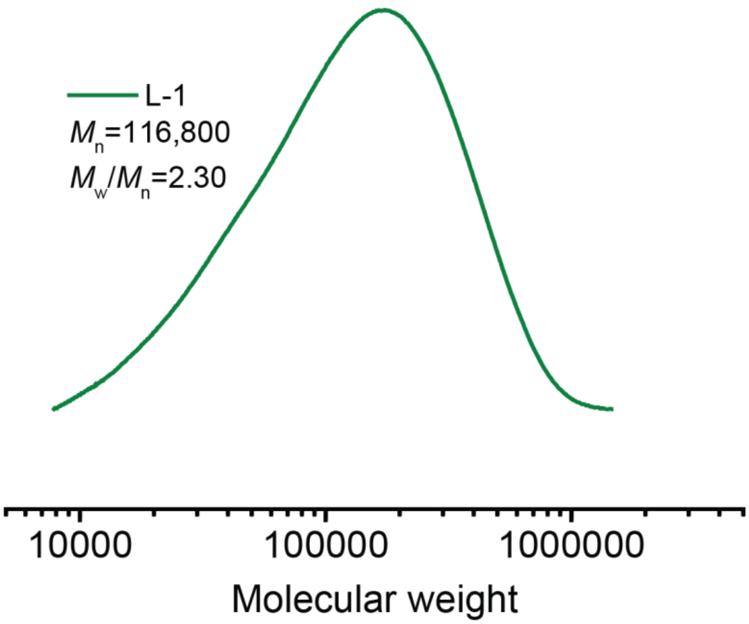

Figure S1. SEC trace of linear PI homopolymers.

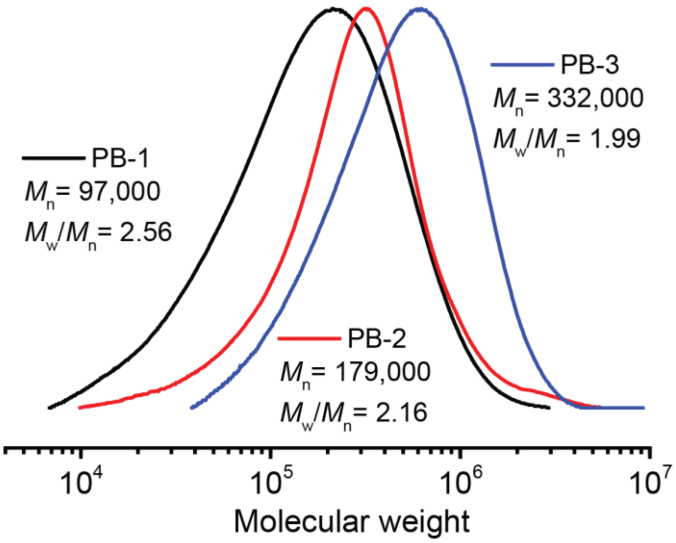

Figure S2. SEC traces of SiO_2-*g*-PI particle brushes.

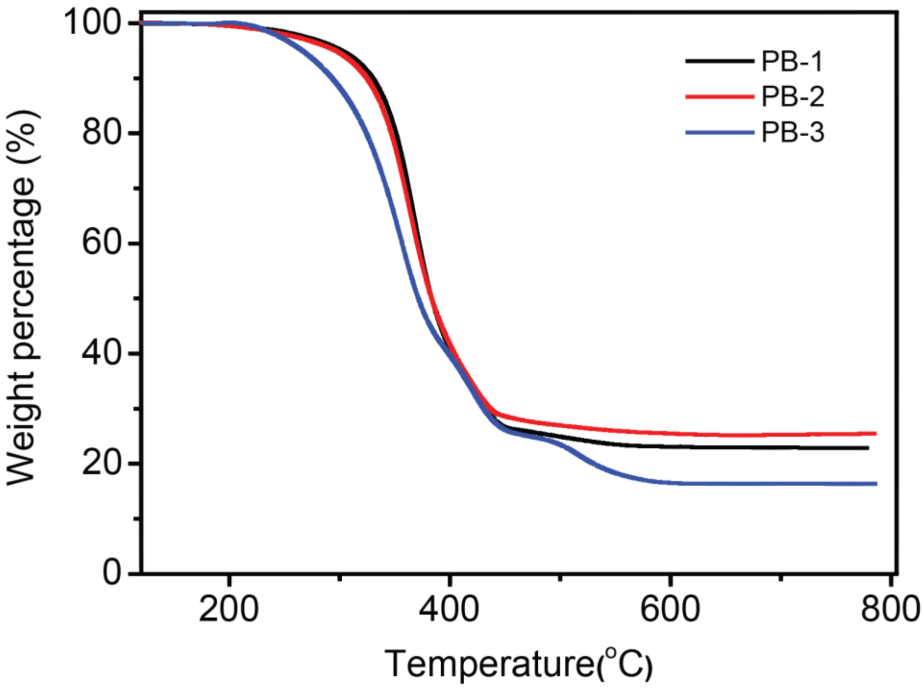

Figure S3. TGA curves of SiO_2-*g*-PI particle brushes.

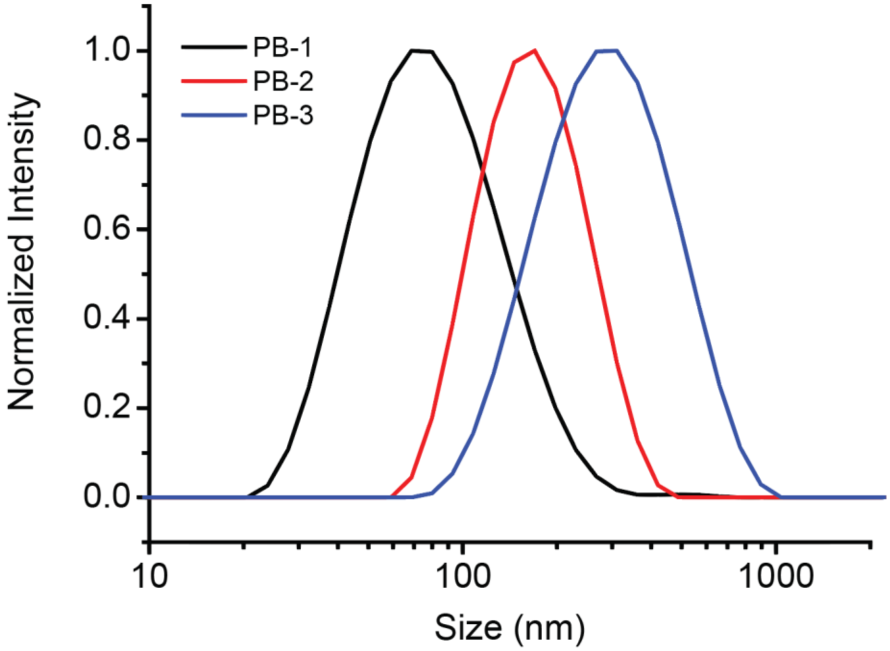

Figure S4. Size distribution of SiO_2-*g*-PI particle brushes measured by DLS in THF solutions. Volume-average particle brush size: PB-1, 72 nm; PB-2, 167 nm; PB-3, 286 nm.

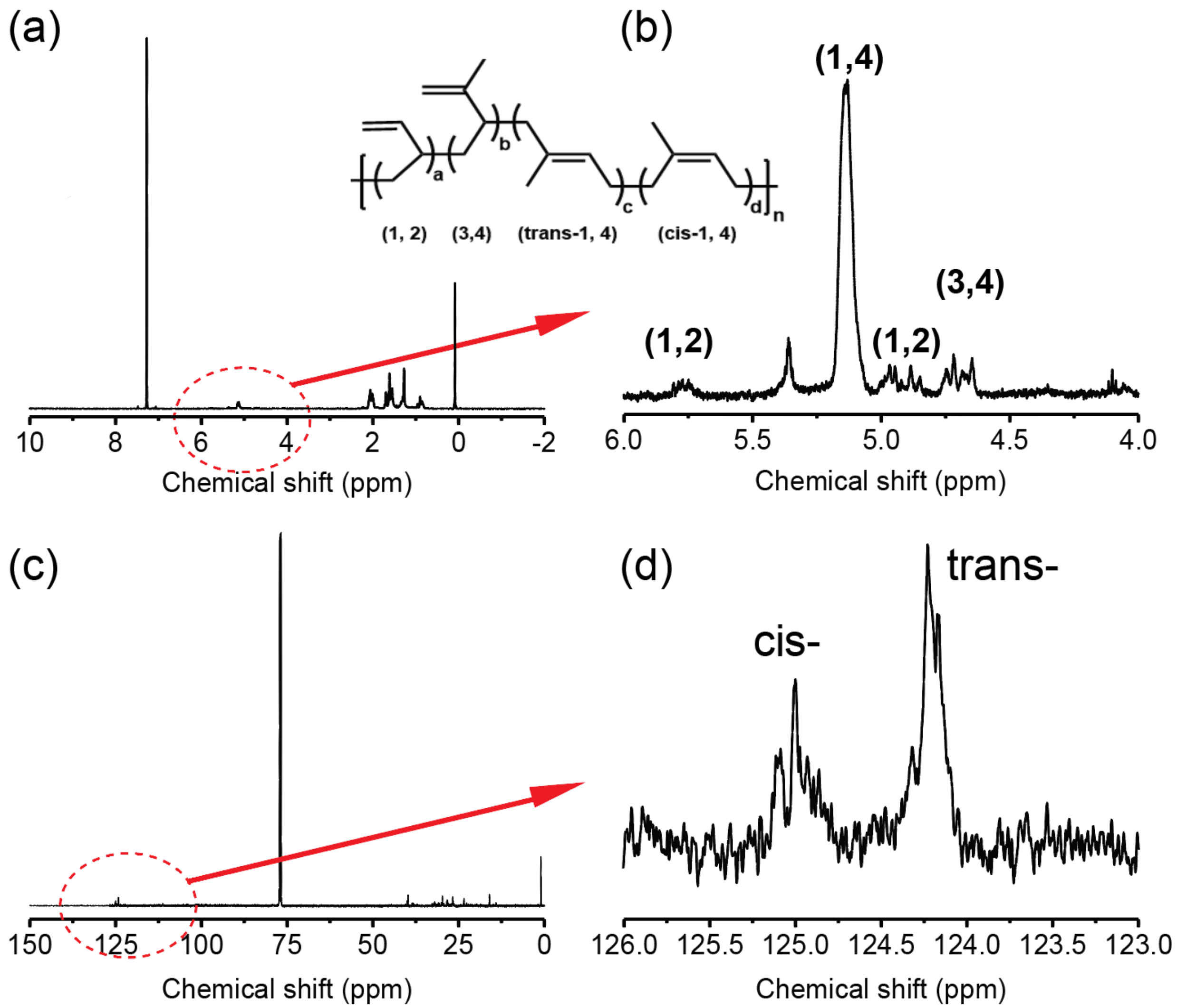

Figure S5. Tacticity of PI synthesized through ATRP. (a) and (b) ^{1}H NMR spectrum of PI (sample: L-1), (c) and (d) ^{13}C NMR spectrum of PI (sample: L-1).

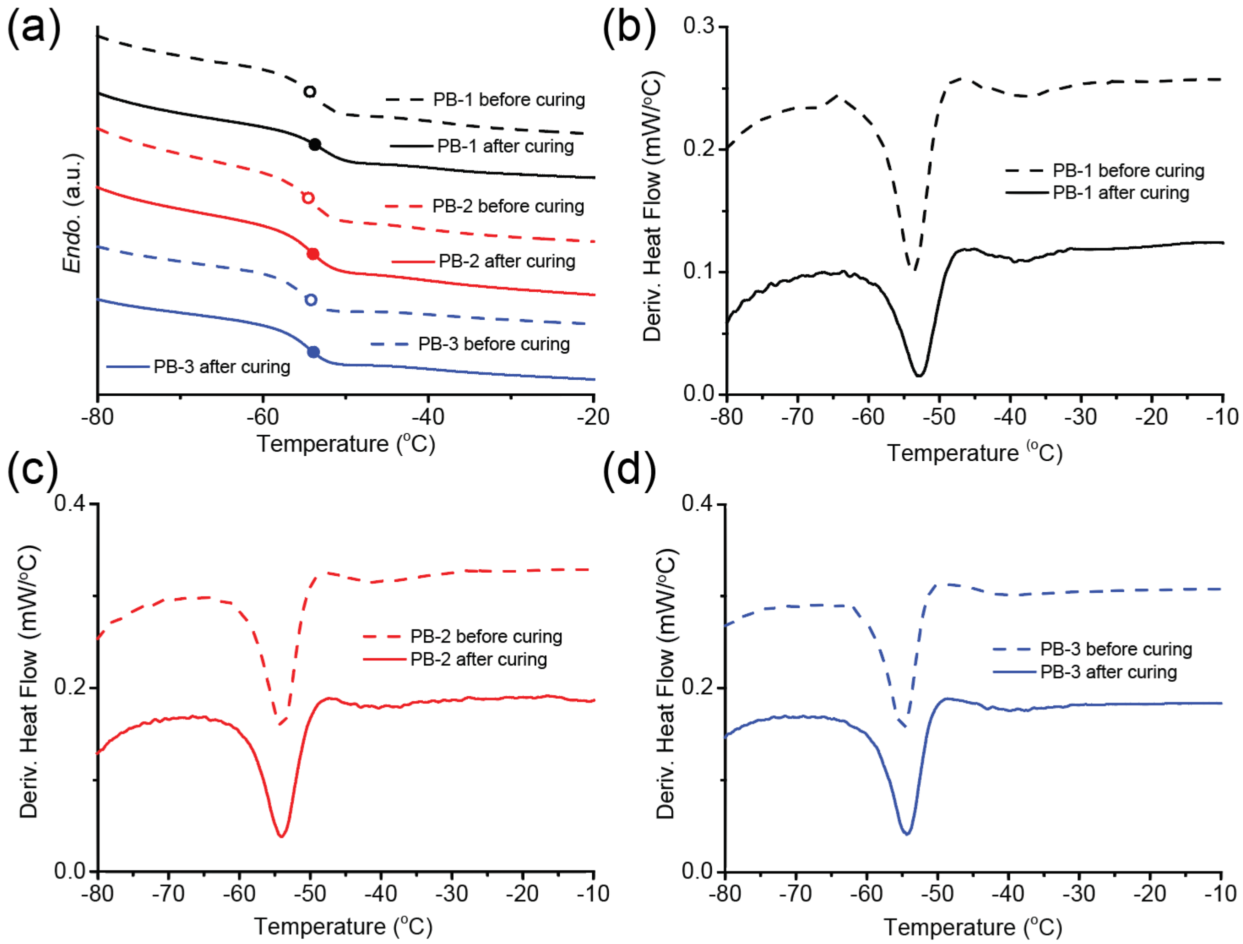

Figure S6. (a) DSC curves of SiO_2-*g*-PI particle brushes before and after curing, (b)-(d) Plot of deriv. heat flow *vs.* temperature of SiO_2-*g*-PI particle brushes before and after curing.

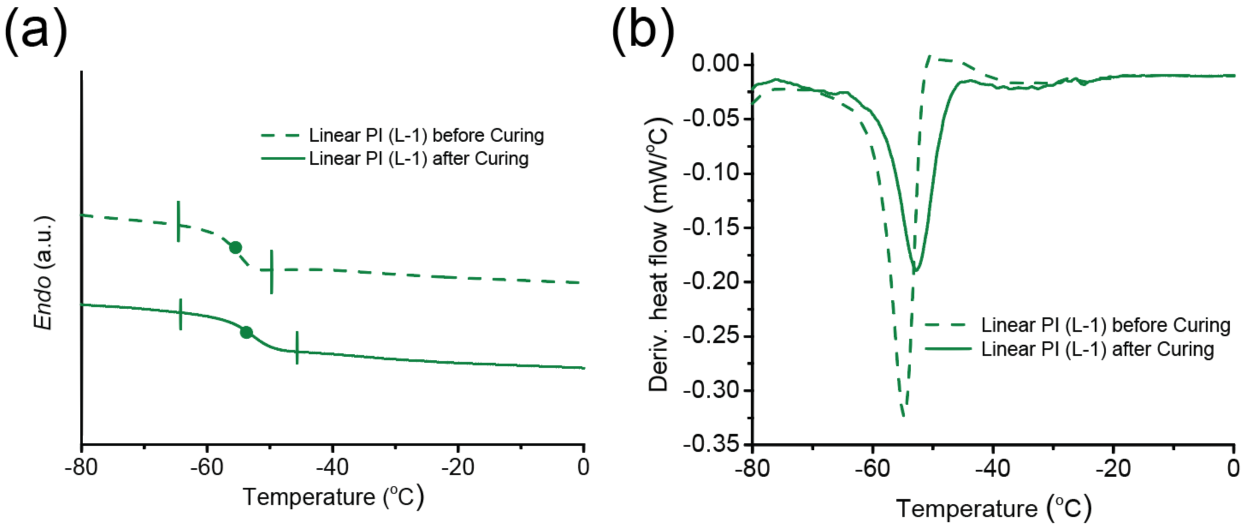

Figure S7. (a) DSC curves of linear PI homopolymers before and after curing, (b) Plot of deriv. heat flow *vs.* temperature of linear PI homopolymers before and after curing.

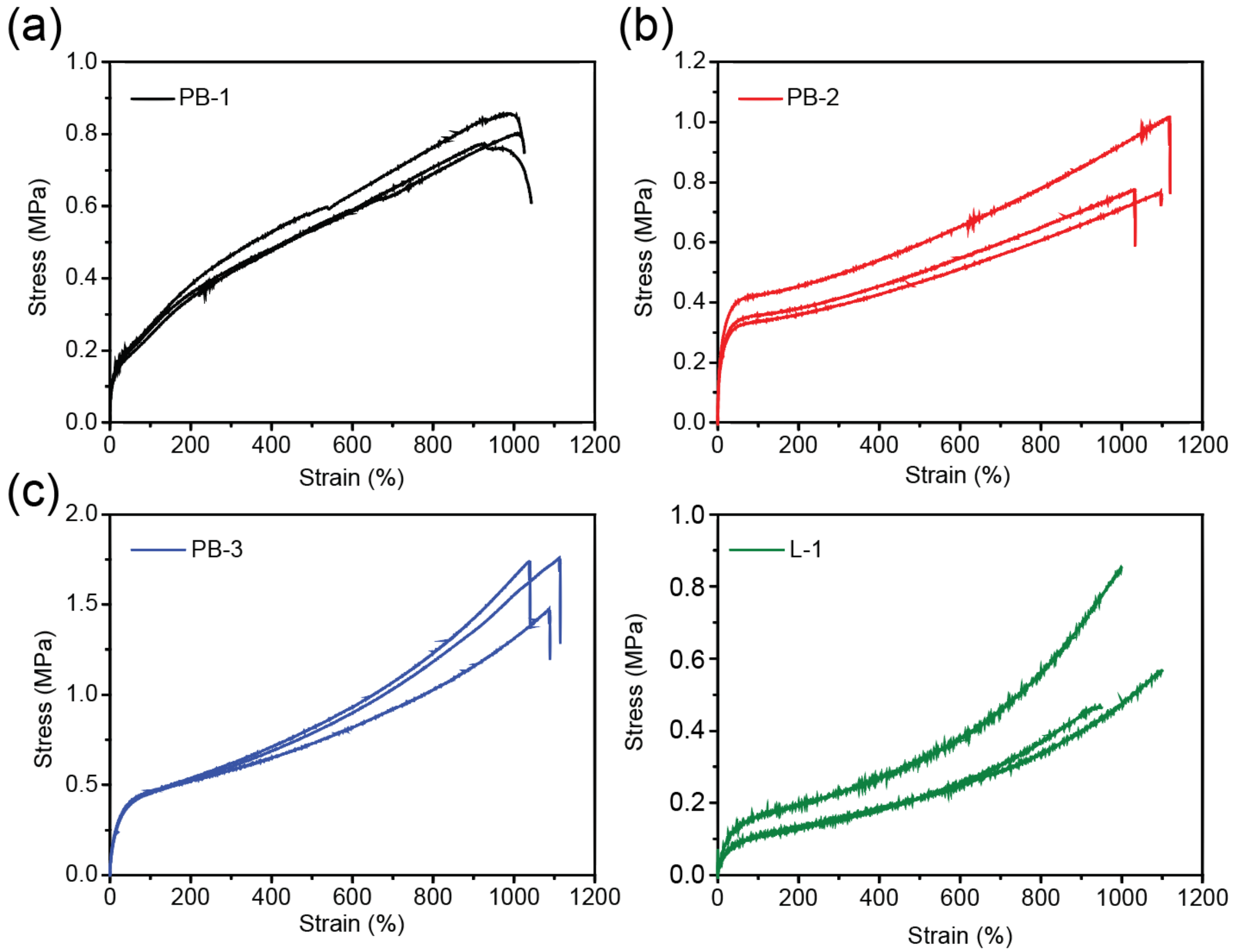

Figure S8. Strain-stress curves of SiO_2-*g*-PI particle brush and linear PI homopolymer bulk films: (a) PB-1, (b) PB-2, (c) PB-3, (d) L-1. All samples were measured three times with different bulk films as shown in different color lines in the figures.

References

1. Matyjaszewski, K.; Miller, P. J.; Shukla, N.; Immaraporn, B.; Gelman, A.; Luokala, B. B.; Siclovan, T. M.; Kickelbick, G.; Vallant, T.; Hoffmann, H.; Pakula, T., Polymers at Interfaces: Using Atom Transfer Radical Polymerization in the Controlled Growth of Homopolymers and Block Copolymers from Silicon Surfaces in the Absence of Untethered Sacrificial Initiator. *Macromolecules* **1999,** *32* (26), 8716-8724.
2. Pyun, J.; Jia, S.; Kowalewski, T.; Patterson, G. D.; Matyjaszewski, K., Synthesis and Characterization of Organic/Inorganic Hybrid Nanoparticles: Kinetics of Surface-Initiated Atom Transfer Radical Polymerization and Morphology of Hybrid Nanoparticle Ultrathin Films. *Macromolecules* **2003,** *36* (14), 5094-5104.
3. Yan, J.; Pan, X.; Schmitt, M.; Wang, Z.; Bockstaller, M. R.; Matyjaszewski, K., Enhancing Initiation Efficiency in Metal-Free Surface-Initiated Atom Transfer Radical Polymerization (SI-ATRP). *ACS Macro Letters* **2016,** *5* (6), 661-665.
4. SMALE, S., Mathematical Problems for the Next Century. In *The Collected Papers of Stephen Smale*, pp 480-488.
5. Hou, G.; Li, S.; Liu, J.; Weng, Y.; Zhang, L., Designing high performance polymer nanocomposites by incorporating robustness-controlled polymeric nanoparticles: insights from molecular dynamics. *Physical Chemistry Chemical Physics* **2022,** *24* (5), 2813-2825.
6. Hou, G.; Tao, W.; Liu, J.; Gao, Y.; Zhang, L.; Li, Y., Tailoring the dispersion of nanoparticles and the mechanical behavior of polymer nanocomposites by designing the chain architecture. *Physical Chemistry Chemical Physics* **2017,** *19* (47), 32024-32037.
7. Hou, G.; Xia, X.; Liu, J.; Wang, W.; Dong, M.; Zhang, L., Designing Superlattice Structure via Self-Assembly of One-Component Polymer-Grafted Nanoparticles. *The Journal of Physical Chemistry B* **2019,** *123* (9), 2157-2168.
8. Sharma, A.; Mukhopadhyay, T.; Rangappa, S. M.; Siengchin, S.; Kushvaha, V., Advances in Computational Intelligence of Polymer Composite Materials: Machine Learning Assisted Modeling, Analysis and Design. *Archives of Computational Methods in Engineering* **2022,** *29* (5), 3341-3385.
